# Trends in alcohol withdrawal management by medical toxicologists in the acute care setting: an analysis of the Toxicology Investigators Consortium (ToxIC) Core Registry, 2016–2022

**DOI:** 10.1093/alcalc/agaf047

**Published:** 2025-07-30

**Authors:** John D DelBianco, Kira J Galeano, Gillian A Beauchamp, Ryan M Surmaitis, Stephanie E Shara, Alison Sutter, Hope Kincaid, Joseph J Stirparo, Kathryn L Wheel, Alexandra M Amaducci, Toxicology Investigators Consortium (ToxIC)

**Affiliations:** Division of Medical Toxicology, Department of Emergency and Hospital Medicine, Lehigh Valley Health Network/USF Morsani College of Medicine, 1200 S. Cedar Crest Blvd., Allentown, PA 18103, United States; Division of Medical Toxicology, Department of Emergency and Hospital Medicine, Lehigh Valley Health Network/USF Morsani College of Medicine, 1200 S. Cedar Crest Blvd., Allentown, PA 18103, United States; Division of Medical Toxicology, Department of Emergency and Hospital Medicine, Lehigh Valley Health Network/USF Morsani College of Medicine, 1200 S. Cedar Crest Blvd., Allentown, PA 18103, United States; Division of Medical Toxicology, Department of Emergency and Hospital Medicine, Lehigh Valley Health Network/USF Morsani College of Medicine, 1200 S. Cedar Crest Blvd., Allentown, PA 18103, United States; Department of Emergency and Hospital Medicine, Lehigh Valley Health Network/USF Morsani College of Medicine, 1200 S. Cedar Crest Blvd., Allentown, PA 18103, United States; Network Office of Research and Innovation, Lehigh Valley Health Network/USF Morsani College of Medicine, 1255 S. Cedar Crest Blvd., Suite 3200, Allentown, PA 18103, United States; Network Office of Research and Innovation, Lehigh Valley Health Network/USF Morsani College of Medicine, 1255 S. Cedar Crest Blvd., Suite 3200, Allentown, PA 18103, United States; Division of Trauma and General Surgery, Institute for Surgical Excellence, Lehigh Valley Health Network/USF Morsani College of Medicine, 1200 S. Cedar Crest Blvd., Allentown, PA 18103, United States; Division of Trauma and General Surgery, Institute for Surgical Excellence, Lehigh Valley Health Network/USF Morsani College of Medicine, 1200 S. Cedar Crest Blvd., Allentown, PA 18103, United States; Division of Medical Toxicology, Department of Emergency and Hospital Medicine, Lehigh Valley Health Network/USF Morsani College of Medicine, 1200 S. Cedar Crest Blvd., Allentown, PA 18103, United States

**Keywords:** alcohol withdrawal syndrome, benzodiazepines, phenobarbital, ketamine, dexmedetomidine

## Abstract

**Aims:**

Alcohol withdrawal syndrome (AWS) requires urgent treatment to prevent morbidity and mortality. In the acute care setting, medical toxicologists play a critical role in AWS management, including the use of gamma-aminobutyric acid agonists and adjunctive medications. We aim to introduce the addiction medicine community to this role by describing clinical presentation and treatment of patients with AWS in the Toxicology Investigators Consortium Core Registry.

**Methods:**

Medical toxicologists from participating sites enter demographic, exposure, clinical presentation, and treatment data on all patients they evaluate into the Core Registry. This was a secondary analysis of registry patients evaluated for AWS from 2016 to 2022. Data were coded in a spreadsheet and analyzed using descriptive statistics.

**Results:**

We included 1093 cases. Agitation and delirium/toxic psychosis were documented in 373 (34.1%) and 227 (20.8%) patients, respectively. Benzodiazepines were the most common gamma-aminobutyric acid agonist treatment (*n* = 539, 49.3%). There was an overall decrease in the use of benzodiazepines alone and increases in the use of phenobarbital, ketamine, and dexmedetomidine. Intubation was performed in 115 (10.5%) patients. Naltrexone, used for alcohol use disorder, was given in 88 (8.1%) cases. The absolute number of AWS cases increased during this period.

**Conclusions:**

Use of benzodiazepines alone to manage AWS decreased while phenobarbital, ketamine, and dexmedetomidine use increased. Many patients had severe withdrawal manifestations, and some received alcohol use disorder treatment, suggesting that medical toxicologists see more severe cases in the acute care setting and have an opportunity to address the underlying use disorder.

## Teaser

**Short Summary:** This study introduces the addiction medicine community to the role of medical toxicologists in the acute management of alcohol withdrawal by describing clinical features and treatment trends in the Toxicology Investigators Consortium Core Registry. Use of benzodiazepines alone decreased while phenobarbital use increased in this overall ill patient population.

## Introduction

According to the World Health Organization, ~400 million people worldwide have an alcohol use disorder (AUD) and there were 2.6 million alcohol-related deaths in 2019 (World Health Organization 2024). Central nervous system dysregulation due to chronic alcohol consumption contributes to dependence and places patients at risk for withdrawal, which can be life threatening (Schuckit 2014). Patients with or at risk for severe alcohol withdrawal syndrome (AWS), which includes seizures, agitation, and autonomic instability, require intensive management in a closely monitored setting.

Medical toxicology is a subspecialty dealing with poisonings, envenomations, environmental and occupational exposures, and adverse effects of medications. It also includes the management of substance use disorders and withdrawal syndromes. Medical toxicologists can assist in the complex care of critically ill AWS patients, who may need continuous cardiorespiratory monitoring, parenteral medications, titrated infusions, and deep sedation with mechanical ventilation.

Although most medical toxicology fellows have a background in emergency medicine, training is open to physicians from all specialties (Pizon et al. 2023). Regardless of their primary specialty, all fellows are required to have experience in the bedside management of patients in the intensive care unit (ICU) (Accreditation Council for Graduate Medical Education 2023). Through the study of neurobiology and pharmacology, fellows develop a detailed understanding of the pathophysiology of intoxication, dependence, and withdrawal, as well as the pharmacologic principles underlying withdrawal management. Fellows are also trained in the identification and treatment of substance use disorders (Hendrickson et al. 2021). Medical toxicologists put this expertise to work in a variety of settings, including inpatient and emergency department (ED) consultation services, inpatient admitting services, and outpatient clinics. In the inpatient setting, most medical toxicologists provide consultation services rather than admitting patients directly. Given their critical care experience, medical toxicologists are well suited to assist with care of patients with severe AWS.

Medical toxicology best treatment practices are constantly being refined. In the case of AWS, benzodiazepines, which agonize the gamma-aminobutyric acid (GABA) type A receptor, are recommended by the American Society of Addiction Medicine as primary pharmacotherapy for AWS (American Society of Addiction Medicine 2020). However, they sometimes fail to achieve adequate syndromic control and have been subject to repeated supply shortages over the past two decades (Whitledge et al. 2023). Recent literature has shown increasing use of the barbiturate phenobarbital to manage AWS, either combined with or in place of benzodiazepines (Goodberlet et al. 2021, Alwakeel et al. 2023, Hundert et al. 2024, Kessel et al. 2024). There has also been greater focus on adjunctive medications to treat severe AWS, including the *N*-methyl-d-aspartate receptor antagonist ketamine and the alpha-2 receptor agonist dexmedetomidine (Mueller et al. 2014, Wong et al. 2014, Linn and Loeser 2015, Pizon et al. 2018, Shah et al. 2018). However, generalizability of the evidence for phenobarbital, ketamine, and dexmedetomidine is limited by the observational nature of the studies.

Understanding the treatment trends in the field of medical toxicology can better inform patient care, especially with acute conditions like AWS. To further characterize the evaluation and management practices of bedside medical toxicologists, the American College of Medical Toxicology (ACMT) established the Toxicology Investigators Consortium (ToxIC), which includes the Core Registry, in 2009. Medical toxicologists at participating sites submit de-identified information to the Core Registry on all patients they have evaluated in the inpatient and outpatient settings for any toxicology-related reason, including envenomation, overdose, adverse medication reactions, and withdrawal (Wax et al. 2011). The number of sites contributing data has changed over time. In 2022, 47 US medical centers across 23 states participated, as well as 5 medical centers in Canada, England, Israel, and Thailand. ACMT manages the Core Registry and performs regular quality assurance reviews (Amaducci et al. 2023).

The primary objective of this study was to characterize trends in AWS management from 2016 to 2022 by medical toxicologists. Specifically, we examined how the pharmacologic management of AWS by medical toxicologists in the acute care setting, including the ED and inpatient units, has changed over time as reflected in the Core Registry. The secondary objective of this study was to describe withdrawal-related signs and symptoms among patients treated for AWS in the Core Registry. Through this analysis focusing on patients with AWS in the acute care setting, we aim to introduce the addiction medicine community to the types of interventions performed by medical toxicologists providing bedside care in this high-risk population.

## Methods

This was a secondary analysis of the prospective ToxIC Core Registry. Medical toxicologists at participating sites enter into the registry de-identified data on demographics, exposure, clinical manifestations, and treatment of patients seen either primarily or in consultation at the bedside, in clinic, or through telemedicine (Amaducci et al. 2023). Participating medical toxicologists are expected to submit data on all patients they evaluate, both in the inpatient and outpatient settings (Wax et al. 2011). The fields on the Core Registry form are required to be completed by the medical toxicologist entering the data, even if the answer is “none” or “not applicable.” There are also free-text fields to enter relevant information not already listed on the form, including specific laboratory values and other treatment or medications such as gabapentin. See Table 1 for a list of the Core Registry variables used in this analysis. ACMT reviews entries as part of a quality assurance process and contacts the submitting institution when data are contradictory or incomplete.

**Table 1:** Demographic and clinical information

**Variable (*n* = 1093)**	***n* (%)**
Age in years, mean ± SD (*n* = 1086)	46.97 ± 12.74
Sex, *n* (%)	
Female	248 (22.7)
Male	843 (77.1)
Transgender	2 (0.2)
Nature of toxicology encounter, *n* (%)	
Attending (patient admitted to medical toxicology service)	223 (20.4)
Consult (bedside consult performed on a patient admitted to a non-medical toxicology service)	870 (79.6)
Outpatient/clinic/office consultation	0 (0)
Clinical features, *n* (%)	
Patient had any signs or symptoms listed in Core Registry	1013 (92.7)
Alcoholic ketoacidosis	99 (9.1)
Tachycardia (HR > 140 beats per minute)	298 (27.3)
Hypertension (SBP > 200 mmHg and/or DBP > 120 mmHg)	121 (11.1)
Hyperthermia (T > 105°F)	5 (0.5)
Hyperreflexia/myoclonus/clonus/tremor	425 (38.9)
Agitation	373 (34.1)
Delirium/toxic psychosis	227 (20.8)
Hallucinations	160 (14.6)
Seizures	163 (14.9)
Thrombocytopenia (platelets <100 thousand/mm^3^)	69 (6.3)
Hepatotoxicity (AST > 1000 U/L and/or ALT > 100 U/L)	45 (4.1)
Pancreatitis (lipase > 1000 U/L)	42 (3.8)
Rhabdomyolysis (creatine kinase > 1000 U/L)	23 (2.1)
Pharmacologic management, *n* (%)	
Neither benzodiazepines nor phenobarbital	91 (8.3)
Benzodiazepines without phenobarbital	539 (49.3)
Phenobarbital without benzodiazepines	191 (17.5)
Both benzodiazepines and phenobarbital	272 (24.9)
Clonidine	27 (2.5)
Dexmedetomidine	21 (1.9)
Ketamine	15 (1.4)
Gabapentin^*^	4 (0.4)
Non-pharmacologic management, *n* (%)	
Intubation/ventilatory management	115 (10.5)
Addiction treatment, *n* (%)	
Acamprosate	17 (1.6)
Disulfiram	1 (0.1)
Naltrexone	88 (8.1)
In-hospital mortality, *n* (%)	
Expired	3 (0.3)
Alive	1085 (99.3)
N/A (outpatient)	4 (0.4)
Missing	1 (0.1)

HR, heart rate; SBP, systolic blood pressure; DBP, diastolic blood pressure; T, temperature; AST, aspartate aminotransferase; ALT, alanine aminotransferase; SD, standard deviation; N/A, not applicable

^*^Gabapentin is not an explicit treatment option to select when entering data into the Core Registry, so all cases of gabapentin included here were entered as “other” treatment

**Figure 1:**
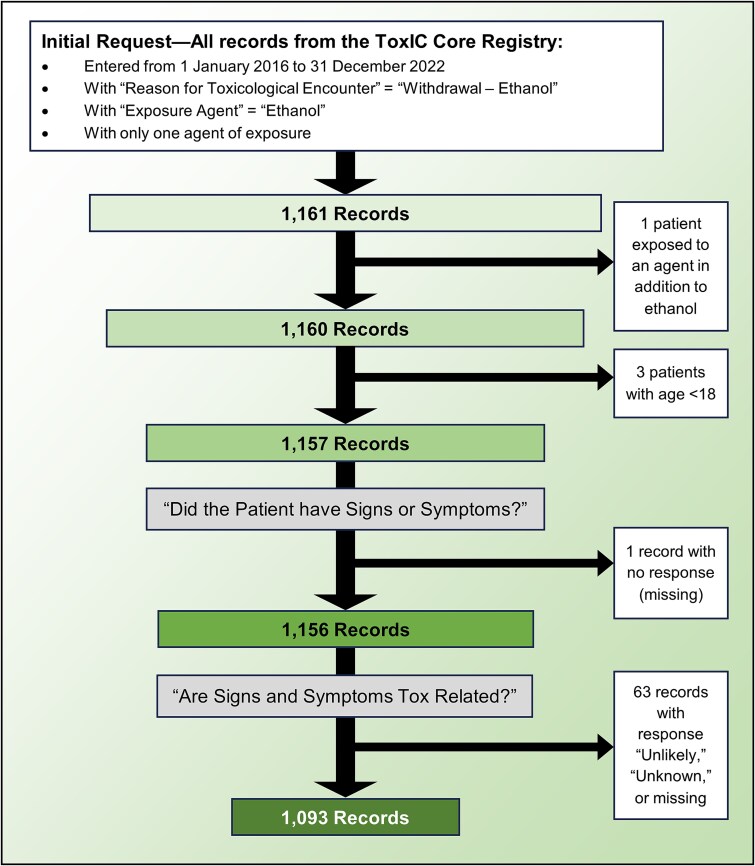
Case selection for inclusion. Flow chart of inclusion criteria, demonstrating which cases were excluded from analysis at each step in the process

All cases submitted to the Core Registry between 1 January 2016 and 31 December 2022 in which AWS was the only reason for treatment by a medical toxicologist were requested from ACMT for use in this analysis. There were substantial data collection changes made to the Core Registry around 2016, so we decided to exclude earlier cases to ensure the data we analyzed had been collected uniformly. Our study ends in 2022 because this was the most recent data available at the time we designed and initiated the study. Data were extracted from the database management system, REDCap (Research Electronic Data Capture) (Harris et al. 2009, Harris et al. 2019), and stored for management and cleaning in a password-protected Microsoft Excel spreadsheet (Microsoft Corporation, Redmond, WA). A flowchart of case selection for inclusion is given in Fig. 1. All cases in the initial dataset had AWS listed as the sole reason for consultation by medical toxicology. We excluded three cases with patients under 18 years of age because AWS management strategies may differ in the pediatric population, and we sought to focus on adult patients in our analysis. Records with unknown exact age were excluded unless the reported age range indicated the patient was 18 years or older. The Core Registry asks the medical toxicologist entering the data to indicate whether the patient had any of the signs or symptoms listed on the form. One case in our initial data was missing a response for this question, so it was excluded. If the toxicologist selects “Yes” to the question about signs and symptoms, they are then asked whether the patient’s signs or symptoms are related to a toxicologic issue, with response options of “Most Likely,” “Unlikely,” or “Unknown.” We excluded 63 records for which “Unlikely” or “Unknown” were selected, or for which no response was given.

After the specified exclusion criteria were applied, we performed descriptive statistical analysis using SPSS Statistics, version 29 (IBM, Armonk, NY). We examined the following variables: the patient’s demographic information, the role of the medical toxicologist (consulted on or primarily admitted the patient), clinical features, and treatment (Table 1). For clinical features, if the medical toxicologist entering data into the registry selects “Yes” to the question about signs and symptoms, they are given a list of clinical findings to choose from. Some of these options have strict parameters, such as tachycardia being defined as heart rate >140 beats per minute. Other clinical features, such as the presence of agitation or delirium, depend on clinical judgment. We focused on signs and symptoms that may be associated with AWS, such as tachycardia or seizures, or with chronic alcohol use, such as thrombocytopenia or hepatotoxicity. Table 1 lists all the signs and symptoms from the Core Registry we included in our study. In the treatment section of the form, medical toxicologists are asked to indicate any toxicological treatment administered to the patient from a list which includes a variety of medications and procedures. The registry does not record the indication, dosage, timing, or number of administrations of the medications. Some treatments, such as benzodiazepines, are only listed by their pharmacologic class, while other medications sometimes used to manage AWS, such as gabapentin, are not included as specific treatment options to select. We focused our analysis on quantifying the use of two GABA agonists, benzodiazepines and phenobarbital, along with the following adjunctive medications: clonidine, dexmedetomidine, ketamine, and gabapentin. Additionally, since AWS is one of the 11 diagnostic criteria for AUD (American Psychiatric Association 2013), medical toxicologists, who receive training in addiction medicine, may see this as an appropriate time to discuss treatment options for AUD with the patient and initiate medication if the patient desires. Thus, we examined three medications used specifically for AUD: acamprosate, disulfiram, and naltrexone. To quantify potential indicators of severe illness, we calculated the frequency of intubation and ventilatory management, as well as the number of patients who died.

The Core Registry is deemed non-human subjects research by a central institutional review board (IRB), the Western Copernicus Group IRB. Participating sites underwent local review with their respective IRBs. Our IRB deemed the current study non-human subjects research.

## Results

After applying the specified exclusion criteria, 1093 cases remained for analysis (Fig. 1). The mean age was 47 years (SD = 13) and 843 (77.1%) were male. More patients were seen in consultation (*n* = 870, 79.6%) than primarily admitted by the treating medical toxicologist (*n* = 223, 20.4%). None of these cases were reported as outpatient consultations. Most patients had signs, symptoms, or laboratory findings related to AWS or chronic alcohol use (*n* = 1013, 92.7%) (Table 1). The most common vital sign abnormality reported was tachycardia (*n* = 298, 27.3%). The most common physical examination finding was hyperreflexia, myoclonus, clonus, and/or tremor (*n* = 425, 38.9%). Agitation and delirium/toxic psychosis were documented in 373 (34.1%) and 227 (20.8%) patients, respectively. Ninety-nine patients (9.1%) presented with alcoholic ketoacidosis. Regarding treatment, the most common GABA agonist used was benzodiazepines (*n* = 539, 49.3%) and clonidine was the most common adjunctive medication (*n* = 27, 2.5%). Of the three medications for AUD that we examined, naltrexone was used most often (*n* = 88, 8.1%). Intubation and mechanical ventilation were performed in 115 (10.5%) patients, and 3 (0.3%) patients died. Demographic, clinical presentation, and treatment information relating to the overall cohort are summarized in Table 1.

**Figure 2:**
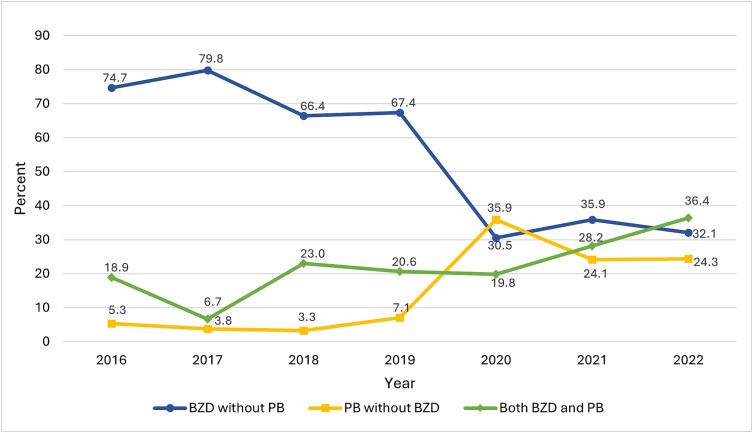
GABA agonist trends. Percentage of patients from each year who received BZD alone, PB alone, or both BZD and PB as GABA agonist treatment. GABA = gamma-aminobutyric acid, BZD = benzodiazepines, PB = phenobarbital

Regarding trends (Table 2), the annual number of AWS cases reported to the Core Registry increased overall, rising from 95 in 2016 to 280 in 2022. Generally, the percentage of patients who received benzodiazepines without phenobarbital decreased, and the percentage who received phenobarbital, with or without benzodiazepines, increased (Fig. 2). There were also overall increases in the percentages of patients receiving ketamine or dexmedetomidine, as well as naltrexone. Table 3 compares the characteristics of patients receiving benzodiazepines and phenobarbital.

**Table 2:** Trends by year

	2016 *n* (%)	2017 *n* (%)	2018 *n* (%)	2019 *n* (%)	2020 *n* (%)	2021 *n* (%)	2022 *n* (%)
Demographics							
Number of cases	95 (8.7)	104 (9.5)	122 (11.2)	141 (12.9)	131 (12.0)	220 (20.1)	280 (25.6)
Patient age in years (mean ± SD)	48.41 ± 12.19 (*n* = 92)	47.39 ± 12.91 (*n* = 104)	46.94 ± 12.73 (*n* = 121)	46.69 ± 11.17 (*n* = 141)	47.52 ± 13.36 (*n* = 131)	47.85 ± 12.76 (*n* = 217)	45.57 ± 13.25 (*n* = 280)
Treatment							
BZD without PB	71 (74.7)	83 (79.8)	81 (66.4)	95 (67.4)	40 (30.5)	79 (35.9)	90 (32.1)
PB without BZD	5 (5.3)	4 (3.8)	4 (3.3)	10 (7.1)	47 (35.9)	53 (24.1)	68 (24.3)
Both BZD and PB	18 (18.9)	7 (6.7)	28 (23.0)	29 (20.6)	26 (19.8)	62 (28.2)	102 (36.4)
Ketamine	1 (1.1)	1 (1.0)	1 (0.8)	1 (0.7)	1 (0.8)	4 (1.8)	6 (2.1)
DEX	0 (0)	0 (0)	1 (0.8)	0 (0)	1 (0.8)	12 (5.5)	7 (2.5)
Naltrexone*	0 (0)	1 (1.0)	3 (2.5)	5 (3.5)	14 (10.7)	15 (6.8)	50 (17.9)
Intubations	15 (15.8)	11 (10.6)	16 (13.1)	15 (10.6)	13 (9.9)	18 (8.2)	27 (9.6)
Clinical features							
Seizures	16 (16.8)	13 (12.5)	19 (15.6)	20 (14.2)	19 (14.5)	24 (10.9)	52 (18.6)
Delirium/toxic psychosis	34 (35.8)	32 (30.8)	29 (23.8)	36 (25.5)	29 (22.1)	45 (20.5)	22 (7.9)

SD, standard deviation; BZD, benzodiazepines; PB, phenobarbital; DEX, dexmedetomidine

^*^Naltrexone was added to the Core Registry as an explicit treatment option to select in 2018

**Table 3:** Comparison of GABA agonist treatment

	Neither BZD nor PB (*n* = 91)	BZD without PB (*n* = 539)	PB without BZD (*n* = 191)	Both BZD and PB (*n* = 272)
Age (mean ± SD) (*n* = 1086)	48.10 ± 12.24	46.12 ± 13.00	48.22 ± 12.77	47.41 ± 12.28
Sex, *n* (%)				
Male	75 (82.4)	411 (76.3)	136 (71.2)	221 (81.3)
Female	16 (17.6)	126 (23.4)	55 (28.8)	51 (18.8)
Transgender	0 (0)	2 (0.4)	0 (0)	0 (0)
Clinical features, *n* (%)				
Tachycardia	14 (15.4)	171 (31.7)	30 (15.7)	83 (30.5)
Hypertension	13 (14.3)	51 (9.5)	16 (8.4)	41 (15.1)
Hyperthermia	0 (0)	4 (0.7)	0 (0)	1 (0.4)
Agitation	22 (24.2)	197 (36.5)	46 (24.1)	108 (39.7)
Delirium	11 (12.1)	122 (22.6)	22 (11.5)	72 (26.5)
Seizures	7 (7.7)	86 (16.0)	24 (12.6)	46 (16.9)
Adjuncts, *n* (%)				
Ketamine	0 (0)	3 (0.6)	4 (2.1)	8 (2.9)
Dexmedetomidine	1 (1.1)	3 (0.6)	4 (2.1)	13 (4.8)
Clonidine	0 (0)	5 (0.9)	8 (4.2)	14 (5.1)
Non-pharmacologic management, *n* (%)				
Intubation	8 (8.8)	62 (11.5)	14 (7.3)	31 (11.4)
In-hospital mortality, *n* (%)				
Expired	1 (1.1)	1 (0.2)	0 (0)	1 (0.4)
Alive	88 (96.7)	536 (99.4)	190 (99.5)	271 (99.6)
N/A (outpatient)	2 (2.2)	1 (0.2)	1 (0.5)	0 (0)
Missing	0 (0)	1 (0.2)	0 (0)	0 (0)

GABA, gamma-aminobutyric acid; BZD, benzodiazepines; PB, phenobarbital; SD, standard deviation; N/A, not applicable

## Discussion

The absolute number of AWS cases submitted to the Core Registry increased overall during the study period. This may be related to more sites participating in the Core Registry and reporting data over time, although published data indicate that there was no overall increase in the total number of cases reported to the Core Registry during the study period (Farrugia et al. 2017, Amaducci et al. 2023). Furthermore, the large increase in AWS cases from 2020 to 2021 coincided with the COVID-19 pandemic, which has been associated with increased alcohol consumption and higher rates of AWS among hospitalized patients (Pollard et al. 2020, Sharma et al. 2021). Regardless of the cause, AWS appears to be a condition increasingly reported to the Core Registry.

The number of males in our study was more than three times higher than the number of females. This finding is consistent with data showing that alcohol consumption and AUD are more prevalent among males (White 2020, World Health Organization 2024). However, this gender difference is decreasing across all ages, with data showing less alcohol use among young males and more alcohol use among adult females in the USA. In recent years, ED encounters pertaining to alcohol use have also risen more among females than males (White 2020). The receding gender gaps relating to both alcohol use and its adverse effects highlight the need for more research in this area.

In the cases we examined, most patients received benzodiazepines without phenobarbital from 2016 to 2019, but most from the latter part of the study period, between 2020 and 2022, were managed with an approach incorporating phenobarbital. In fact, phenobarbital was the sole GABA agonist for an increased proportion of patients from 2020 to 2022 relative to the 2016 to 2019 study period (Fig. 2). This trend may be multifactorial, with contributing factors potentially including benzodiazepine shortages and increased awareness of phenobarbital as an alternative or adjunct to benzodiazepines. Phenobarbital, used alone or in addition to benzodiazepines, has been associated with shorter hospital and ICU length-of-stays, less delirium, fewer ICU admissions and intubations, and less use of adjunctive medications compared to benzodiazepines alone (Rosenson et al. 2013, Tidwell et al. 2018, Hawa et al. 2021, Alwakeel et al. 2023, Malone et al. 2023, Hundert et al. 2024). Phenobarbital also has a longer half-life (53 to 140 h) than the long-acting benzodiazepine diazepam (30 to 56 h) (Schmidt et al. 2016). The longer half-life of phenobarbital offers a potential advantage in the outpatient management of AWS in ED patients (Lebin et al. 2022, Pistore et al. 2022). Phenobarbital’s long half-life is a potential safety concern in patients discharged directly from the ED, but this strategy appears to be safe when used as part of a structured protocol (Ebeling-Koning et al. 2024).

Regarding adjunctive medications, ketamine and/or dexmedetomidine were given to only 33 patients (3.0%) in this study, but use of both adjuncts generally increased over the seven years examined. As with phenobarbital, this trend among cases reported to the Core Registry may reflect increasing awareness of the potential benefit of these medications in patients with severe withdrawal. Ketamine has been linked to shorter ICU length-of-stay, and both ketamine and dexmedetomidine have been associated with less benzodiazepine use in patients treated for AWS (Mueller et al. 2014, Wong et al. 2014, Linn and Loeser 2015, Pizon et al. 2018, Shah et al. 2018). Clonidine, which is an alpha-2 receptor agonist similar to dexmedetomidine, was administered to more patients than dexmedetomidine in the data we examined. Clonidine has been incorporated as a routine intervention in some AWS treatment protocols. It is administered by the oral or transdermal routes and therefore, unlike dexmedetomidine, does not require ICU admission and may be an appropriate adjunctive treatment in less severe cases (Smith et al. 2022, McCullough et al. 2024). Most patients receiving ketamine or dexmedetomidine in our study were not intubated. One prior study found that adjunctive ketamine may reduce the need for intubation in patients with AWS, noting that ketamine is unlikely to cause respiratory depression with appropriate dosing (Pizon et al. 2018). We also examined use of gabapentin, another common adjunctive medication. Only four patients were reported to have received gabapentin, so there was an insufficient number to determine a trend. However, this value is likely underreported since gabapentin is not an explicit treatment option to select in the Core Registry. It is important to remember that these adjunctive medications do not agonize GABA receptors and there is a risk of worsening withdrawal especially in inadequately treated patients whose adrenergic symptoms are masked by dexmedetomidine (Wong et al. 2015).

A small but notable number of patients in our study were given medications to treat AUD. Naltrexone, the most used, was increasingly prescribed over the study period. Medical toxicologists routinely manage substance use disorders by initiating or continuing medication, including naltrexone, in both the inpatient and outpatient environments (Laes 2016). Although the medical toxicology evaluation in the acute care setting is focused on treating withdrawal, addressing the underlying use disorder during this encounter is crucial because of the risk of worsening withdrawal on re-admission due to the so-called kindling effect (Becker 1998). Increasing use of naltrexone in our study suggests that there has been a growing focus on treating AUD in patients hospitalized for withdrawal in the Core Registry. Initiation of medication for AUD and referral to outpatient resources for continued care are important interventions that can be implemented in the ED.

It is unclear how many patients in the data examined were admitted to the hospital during the medical toxicology encounter. When asked to describe the nature and location of the medical toxicology encounter, none of the cases we included selected the option to indicate that the patient was seen as an outpatient consultation. However, for the question regarding in-hospital mortality, four cases chose the response indicating this question was not applicable because the patient was not admitted (i.e. outpatient). It is plausible that at least some of these patients were discharged from the ED and therefore not considered to have been hospitalized. Despite these few inconsistencies, it is clear that the patients included in our study were overall ill, with many requiring intubation or experiencing withdrawal-related psychosis or delirium, hallucinations, and seizures. This suggests that medical toxicologists tend to see more severe cases of AWS in the acute care setting. There were no identifiable trends in intubations or seizures over the study period. There was an overall decrease in the number of reported cases involving delirium/toxic psychosis. Future research should determine if this trend remains with larger numbers of patients, and if there is any correlation between decreased delirium and greater focus on early and aggressive AWS treatment.

Our study has several limitations. To start, the scope of our analysis is limited by the available data. The Core Registry does not record a patient’s primary reason for admission, which may differ from the reason for medical toxicology consultation. A patient’s clinical course may be complicated by conditions unrelated to AWS. In cases involving patient death, the reason for death is not collected. There may have been comorbidities or concurrent illnesses relevant to a patient’s death that are not reported in the Core Registry. Because it is unknown to what extent alcohol use or withdrawal contributed to the deaths, it would be difficult to draw any conclusions from these cases. Some variables, such as race, ethnicity, or inpatient location (including ED, hospital floor, and ICU), were not included in our analysis because many records were missing this information.

As previously mentioned, the Core Registry has strict parameters for some variables, such as tachycardia being defined as a heart rate >140 beats per minute. Therefore, patients with no documented signs or symptoms are not necessarily asymptomatic, but rather may not have had symptoms, vital signs, or laboratory values meeting the Core Registry parameters. Conversely, some of the signs and symptoms, and their most likely cause, rely on the clinical judgment and expertise of the medical toxicologist examining the patient and reporting the data. Furthermore, data entry errors are possible.

Treatment data are specifically limited in the Core Registry in several ways. Only treatment related to the patient’s toxicologic problem, which in this case is AWS, is entered into the Core Registry. The registry does not record treatments unrelated to the toxicological encounter, or the indication, dosage, timing, or number of administrations of toxicologic treatments. Furthermore, since some treatment options in the registry are only listed by pharmaceutical class, the specific medication or medications administered are unknown. For instance, the Core Registry lists “benzodiazepines” as a treatment option, but several benzodiazepines with varying pharmacokinetic properties are used in clinical practice, so it is unknown which benzodiazepine, or combination thereof, was given in these cases. Gabapentin is not an explicit treatment option to select when entering data into the Core Registry, so its use is likely underreported. Naltrexone, acamprosate, and disulfiram are also likely underreported because they were only added to the Core Registry in 2018. In addition, it is unknown if these AUD medications were started during the patient’s hospitalization or a continued home medication. Aside from medical interventions such as intubation, the Core Registry does not contain any information on non-pharmacologic AWS treatment.

Our analysis is also limited by our study criteria. Patients in withdrawal from multiple substances, such as alcohol and opioids, or who have concomitant substance use disorders, may present more challenges and benefit most from a medical toxicology evaluation. However, we chose to examine records in which AWS was the only reason for toxicology consultation, as determined by a bedside medical toxicologist. While the study team felt this would reduce the potential for confounding variables and clinical scenarios (such as withdrawal or intoxication related to other substances), we recognize that excluding such cases from this study may have introduced some bias in our analysis and limits generalizability of our results. We did not collect information on AWS cases involving other substances or reasons for medical toxicology evaluation, so the data presented here are not representative of all AWS cases in the Core Registry. Additionally, we focused only on medications pertinent to the treatment of AWS, and patients may have received other medications that we did not analyze. Inferential statistical tests were not conducted for this analysis because the goal was to describe global trends in management.

Finally, the generalizability of our study to other settings is limited. All patients included were treated at sites participating in the Core Registry, many of which are tertiary-care centers with medical toxicology services, so the results cannot be generalized to the ambulatory setting. Generalizability is also limited by the potential for a geographical bias. The trends we report reflect practice patterns at the centers contributing data and cannot be used to infer broader trends. The Core Registry includes international sites, and practice variation may be especially pronounced among different countries. However, location cannot be accounted for in our analysis because the locations and identities of contributing sites are not disclosed when data from the Core Registry are provided for research.

## Conclusions

We examined the treatment of AWS by medical toxicologists in the acute care setting as reported in the ToxIC Core Registry. The number of AWS cases reported to the Core Registry generally increased from 2016 to 2022. This cohort was collectively ill, suggesting that medical toxicologists in the acute care setting are involved in more severe cases of AWS, which may require intensive monitoring and careful titration of high-risk medications. Overall, benzodiazepines were the most common GABA agonist and clonidine was the most common adjunctive medication used. Over the study period, use of benzodiazepines alone to manage AWS decreased, while use of phenobarbital, either alone or in combination with benzodiazepines, increased. We also saw an overall increase in ketamine and dexmedetomidine use during the study period. A notable number of patients also received medication for AUD, highlighting the role medical toxicologists have in the management of AUD in the acute care setting and the importance of addressing the underlying use disorder while treating the acute withdrawal syndrome.

AWS is a common and potentially life-threatening condition that deserves continued investigation. Considering recent medication shortages, it is more crucial than ever for clinicians to understand all the available treatment options. Larger studies comparing benzodiazepines and phenobarbital on patient-centered outcomes are needed to inform the development of evidence-based protocols. Similarly, future research should examine which patients benefit most from adjunctive medications, including ketamine and dexmedetomidine. That no single GABA agonist or adjunctive medication dominated the others in the data we examined emphasizes the importance of providing individualized treatment to each patient, guided by the clinician’s experience, institutional protocols, and regional preferences. The insight medical toxicologists provide regarding complex pharmacologic management is especially important in critically ill patients, and a medical toxicology consultation, either through a bedside consultation service or regional poison center, should be considered in these challenging cases.

## Data Availability

The data underlying this article were provided by the Toxicology Investigators Consortium (ToxIC) by permission. Data will be shared on request to the corresponding author with permission of ToxIC.
